# Supplementary material for: Is image-to-image translation the panacea for multimodal image registration? A comparative study
Source: PLoS One. 2022 Nov 28;17(11):e0276196. doi: 10.1371/journal.pone.0276196 (PMC9704666; doi:10.1371/journal.pone.0276196)
Supplement: S1 Appendix — (PDF) [file pone.0276196.s001.pdf]

S1 Appendix. List of abbreviations (and method names) used in the paper:

α-AMD	- α-cut based Average Minimal Distance
ADAM	- ADAptive Moment estimation
ASGD	- Adaptive Stochastic Gradient Descent
BF	- Brightfield (microscopy)
CC	- Cross-Correlation
cGAN	- conditional Generative Adversarial Network
CoMIR	- Contrastive Multimodal Image Representation for registration [29]
CurveAlign	- registration method designed for BF and SHG images [28]
CycleGAN	- Cycle-consistent (Generative) Adversarial Network [14]
DCNN	- Deep Convolutional Neural Network
DRIT++	- Diverse Image-to-Image Translation via Disentangled Representations [17]
FID	- Fréchet Inception Distance
GAN	- Generative Adversarial Network
GPU	- Graphics Processing Unit
I2I	- Image-to-Image (translation)
InfoNCE	- (Info) Noise-Contrastive Estimation
MI	- Mutual Information
MIND	- Modality Independent Neighbourhood Descriptor

MSD	- Mean Squared Difference
MR	- Magnetic Resonance
NGF	- Normalised Gradient Fields
NIR	- Near-Infrared
ORB	- Oriented FAST (Features from Accelerated Segment Test) and rotated BRIEF (Binary Robust Independent Elementary Features)
pix2pix	- Image-to-Image Translation with Conditional Adversarial Networks [13]
px	- pixel
QPI	- Quantitative Phase Imaging
RANSAC	- RANdom SAmple Consensus
RGB	- Red-Green-Blue (color model)
RIRE	- Retrospective Image Registration Evaluation (dataset)
SGD	- Stochastic Gradient Descent
SHG	- Second Harmonic Generation
SIFT	- Scale Invariant Feature Transform
SSD	- Sum of Squared Differences
StarGANv2	- Diverse Image Synthesis for Multiple Domains [15]
TMA	- Tissue micro-array
