# Supplementary material for: Is image-to-image translation the panacea for multimodal image registration? A comparative study
Source: PLoS One. 2022 Nov 28;17(11):e0276196. doi: 10.1371/journal.pone.0276196 (PMC9704666; doi:10.1371/journal.pone.0276196)
Supplement: Attachment — Submitted filename: reviewer-comments.pdf [file pone.0276196.s002.pdf]

Is image-to-image translation the panacea for multimodal image registration? A comparative study

Review Comments to the Author

This work tackles the well-known problem of multimodal registration, which lacks proper definitions for similarity functions between aligned images. Recently, due to the advent of many image-to-image translation methods, several methods propose to cast this multimodal registration problem as a monomodal problem, where several similarity functions have proved successful, by means of synthesising source domain images following the appearance of the target domain images.

The authors provide a nice trade-off between some of the standard metrics designed for multimodal registration and some methods using the intermediate I2I step with a monomodal similarity metric. Moreover, they add their recently proposed methods CoMIR, which in my opinion could be though also as an I2I but to a latent domain between the source and target domains.

The methods, datasets and experiments are well presented in the manuscript, even though sometimes it may be confusing due to the wealth of results and comparisons.

However, I have some major comments and some minor comments, both detailed below in a section-by-section basis. Before that, I wanted to highlight my main concern (repeated below). In my opinion, the experiments design may need a bit more of discussion or refactoring. I think that some readers may eventually say that the comparison is not entirely faire unless clarified by the authors. The I2I methods have been trained using different training data (not datasets, but different inputs, for example, aligned vs unaligned images), or hyperparameters (batch size, number of iterations). I acknowledge that all parameters cannot be set equally, but for a fair comparison one needs to decide which ones to keep and which not and justify this choice. In the case of this article, I think that the same training data (all methods sees the same **aligned** pairs in training), and batch size need to be kept constant for all methods. In contrast, the number of training iterations/epoch may be different based on the convergence of the methods (rather than a fixed number of epochs as it is now; in such case, I'd say it's better to fix the same number of epochs for all methods).

Such decisions, independent of which ones, need to need properly justified. The authors mention all these experimental decisions but the reasons behind are not convincing to me.

Background section:
I think that this paper (https://doi.org/10.1007/978-3-642-40811-3_79) asked a similar question about 10 years ago and may be mentioned in the introduction, as a very similar type of work. It can be also interesting to compare the conclusions from that article with the present in the conclusions

The distinction between intensity- and feature-based and hybrid registration methods seems appropriate for this work, since they are mainly focused on the multimodal scenario. They discuss several intensity-based and a few feature-based approaches. In the last paragraphs, they highlight several works that use I2I for registration of medical images. For completeness, I think that this part lacks:

- In which category of the classification introduced by the authors do the I2I methods fall? (intensity, feature, hybrid).
- Why do we need I2I? (justification)
- What are the problems when training I2I translation methods? How is it addressed in the literature? (e.g., in 49 they use invariance between registration and translation)

Moreover, I think there are two concepts probably misused (happy to discuss with the authors so that they can convince me):

- "Monomodal registration frameworks". I don't think a registration framework is monomodal or multimodal per se. It is the metric optimised that is more appropriate for monomodal or multimodal scenarios.
  - As an example, NiftyReg can use the SSD, LNCC or NMI metrics depending on the problem.
  - The authors define the MIND optimisation framework as a "similarity-based (monomodal) registration framework", where they use the SSD between MIND representations of both moving and reference images, even though it can be used to align both monomodal and multimodal images.

- In the same line, I'm not sure that "monomodal registration is easier", there are just better defined losses (intensity-based, voxel-level) than in the multimodal case. For example, SSD of Local NCC.

Considered methods:
I think the second paragraph should be in the results section (less confusing for the reader). You actually mention it later as well.

- "We also evaluated VoxelMorph [32] using MI as a loss function for the task of rigid multimodal registration. However, it consistently under-performed in our rigid registration task (similar is also observed by [57]) and we exclude the related results for clarity."

Moreover, you could in few words say which VoxelMorph configuration did you test: SVF, deformation fields, rigid transform parameters (if exists)?

Experiments:
- Fair comparison of I2I methods: the same data augmentation is used (great!) but:
  - The data used is different! (depending on the need of aligned or not images).
  - The batch sizes are also different.
  - The number of iterations are also different and it is hard coded (it could be justified by convergence for this certain problem, but not regarding the default number of epochs, which are probably optimised for other tasks).

  My thinking is that one could optimise each method and it may be right (e.g., run until convergence), but for a comparison to be fair some training parameters must be fixed and equal for all methods (e.g., the most restrictive method sets the parameter). The criteria should be clear and justified.
  My special concern is with the use of aligned and not aligned training data, especially because training with aligned images seems more appropriate for the task at hand (which is registration, not I2I). Also, the method (at least for DRIT++ is made explicit but I think it's also the case for CycleGAN, StarGANv2), is is trained with unpaired images but tested with paired images. And then in the results sections, CoMIR and Pix2Pix are the best performing ones.

- The feature-wise transformation los DRIT++ should be briefly summarised in the methods section (3.2.3.) as you mention a slight modification and seems important for understanding the method comparison.

Results:
Figure 8 needs indexing: A,B, etc…

Conclusions:
One of the problems with I2I methods is that the topology of the generated image may not be preserved. Then, recent works such as (*https://doi.org/10.1007/978-3-030-87592-3_5., citation 49 and even the authors' method CoMIR could be thought as an being on this line*) propose

different options to ameliorate this fact. I think that, at least, this needs to be discussed somewhere in the paper (maybe linked to the previous comment about “problems with I2I translation methods”?). Maybe not in the conclusions but in sections 6.2 or 6.4.

Minor comments:

1. In the introduction (lines 52-55) the authors seem to say that multimodal image registration using I2I and GAN-based approaches are mostly inexistent. I wouldn’t say so, and even the authors discuss many approaches in the following section.

2. In Fig.1, I would not say that the registration from generated modality B to acquired modality B is simpler (same for modality A), but it’s **monomodal**. Since it is a monomodal registration, you could use standard pixel-based similarity metrics that are well defined in such scenarios. In the case of registering acquired modality A and B, the registration is not harder but **multimodal**; in this case there are not well-defined objective functions to optimise. The most widely used, as you say throughout the text, is the mutual information, but the performance is far from the monomodal scenarios. Moreover, what you depict here is more like a CycleGAN-based approach. I think it would be more clear to the reader just to show a forward transformation (or clarify it in the text and caption).
3. Good to have published the methods open-source in Github. I think it would be useful to have the link in the text (and not only in the abstract). Either in the introduction when you mention it, or in the Methods, section.

4. Please, uniform StarGANv2 and StarGAN-v2 naming convention.

5. I think that MSD acronym is defined after the first use.
